# Racial differences in long-term adherence to oral antidiabetic drug therapy: a longitudinal cohort study

**DOI:** 10.1186/1472-6963-9-24

**Published:** 2009-02-07

**Authors:** Connie M Trinacty, Alyce S Adams, Stephen B Soumerai, Fang Zhang, James B Meigs, John D Piette, Dennis Ross-Degnan

**Affiliations:** 1 Department of Ambulatory Care and Prevention, Harvard Medical School and Harvard Pilgrim Health Care, Boston, MA, USA; 2 Division of Research, Kaiser Permanente Northern California, Oakland, CA, USA; 3 General Medicine Unit, Department of Medicine, Massachusetts General Hospital and Harvard Medical School, Boston, MA, USA; 4 VA Center for Clinical Management Research and Division of General Medicine, University of Michigan Health Care System, Ann Arbor, MI, USA

## Abstract

**Background:**

Adherence to oral antidiabetic medications is often suboptimal. Adherence differences may contribute to health disparities for black diabetes patients, including higher microvascular event rates, greater complication-related disability, and earlier mortality.

**Methods:**

In this longitudinal retrospective cohort study, we used 10 years of patient-level claims and electronic medical record data (1/1/1992–12/31/2001) to assess differences in short- and long-term adherence to oral antidiabetic medication among 1906 newly diagnosed adults with diabetes (26% black, 74% white) in a managed care setting in which all members have prescription drug coverage. Four main outcome measures included: (1) time from diabetes diagnosis until first prescription of oral antidiabetic medication; (2) primary adherence (time from first prescription to prescription fill); (3) time until discontinuation of oral antidiabetic medication from first prescription; and (4) long-term adherence (amount dispensed versus amount prescribed) over a 24-month follow-up from first oral antidiabetic medication prescription.

**Results:**

Black patients were as likely as whites to initiate oral therapy and fill their first prescription, but experienced higher rates of medication discontinuation (HR: 1.8, 95% CI: 1.2, 2.7) and were less adherent over time. These black-white differences increased over the first six months of therapy but stabilized thereafter for patients who initiated on sulfonylureas. Significant black-white differences in adherence levels were constant throughout follow-up for patients initiated on metformin therapy.

**Conclusion:**

Racial differences in adherence to oral antidiabetic drug therapy persist even with equal access to medication. Early and continued emphasis on adherence from initiation of therapy may reduce persistent racial differences in medication use and clinical outcomes.

## Background

Diabetes is a leading cause of death and disability in the United States, costing an estimated $174 billion in lost productivity and health care expenditures each year. [1] Black diabetes patients experience higher rates of microvascular events, greater complication-related disability, and nearly 30% higher mortality than white diabetes patients. [2,3] Landmark randomized clinical trials have demonstrated that intensive drug therapy can improve glycemic control, reduce risk of microvascular and other diabetes-related complications, and reduce overall health care costs. [4-7] Yet, despite the proven benefits of adhering to a prescribed medication regimen, many patients fall short of adherence targets. [8-11]

Racial differences in medication adherence may reflect disparities in other components of diabetes care such as patient education and counseling, insurance coverage, and geographic access to health care services. [12-16] In previous studies, we found that blacks have higher glycemic levels [17] and worse adherence to glucose self-monitoring practice [18,19] even in a setting where variations in the quality of care and insurance coverage have been minimized. Evidence regarding the role of race in predicting adherence to diabetes medications over time has been limited to studies with small sample sizes, too few black patients, and inadequate research designs to assess self-care behavior (e.g., the use of self-report adherence measures). [12-14,20,21] In this longitudinal study, using ten years of clinical and pharmacy claims data and a repeated measures design, we examine the relationship between race and both short- and long-term adherence to medication use among patients newly prescribed an oral antidiabetic drug therapy in an HMO serving a large population of both black and white patients with diabetes.

The purpose of this study was to determine whether racial differences in short-term and long-term adherence exist in this setting where a strong focus is placed on preventive services, coordination of care, and standardization of services and where other possible determinants of suboptimal adherences to medication have been reduced, including economic barriers in access to care. Given our previous findings that racial differences in glycemic control and self-monitoring of blood glucose exist and persist in spite of equal access to services and treatment quality, we expected to find similar race-related differences in medication adherence, with blacks hypothesized to be consistently less adherent to prescribed regimens than whites over time.

## Methods

### Study setting and data sources

Harvard Vanguard Medical Associates (HVMA) is a large multi-specialty care group comprised of 14 health centers serving over 300,000 people of diverse ethnic and socioeconomic backgrounds in the Boston area. Using HVMA's electronic medical record system, we captured patient-level data from 1992–2001 from all ambulatory and inpatient encounters, including pharmacy encounters and laboratory test results, diagnoses, procedures, and therapies. The electronic medical record system also contained data on a patient's date of birth, gender, months of membership, and census tract of residence (which was linked to socioeconomic characteristics of the neighborhood such as income and education using the 1990 census files). In addition, all records for emergency room visits, hospitalizations, and other services received outside the HVMA system were recorded in a linked claims database. Prescriptions for oral antidiabetic medications were extracted from HVMA electronic ambulatory encounters and linked to corresponding pharmacy medication dispensing claims data. Previous studies have documented the reliability of these longitudinal data. [22,23]

### Study population

To be included in the study, patients had to be at least 18 years old, of black or white race, and newly diagnosed with diabetes. Race, the key independent variable, was determined from clinician reports. Although information on race was not uniformly collected, a study comparing self-reported and medical record data on race classification found a 96% agreement (unpublished data). Patients were classified as newly diagnosed based on a record of ≥ 1 inpatient diagnosis of diabetes [ICD-9 codes 250.XX] or ≥ 2 outpatient diabetes diagnoses, or ≥ 1 prescription or dispensing of insulin or oral antidiabetic medication (i.e., first or second generation sulfonylureas, metformin, meglitinides, thiazolidinedione, and alpha-glucosidase inhibitors), with no previous record of a diabetes diagnosis or medication. Patients had to be continuously enrolled (with no more than 45 days of disenrollment) in HVMA and insured with Harvard Pilgrim Health Care (HPHC) for at least one year prior to and one year after the first prescription of oral antidiabetic medication.

Due to the difficulty in measuring insulin adherence because of the wide variation of insulin dosages across patients, adherence measures for patients on insulin treatment were not developed. Patients who were prescribed or dispensed an insulin therapy at any point in the follow-up period were excluded from the study cohort. We also excluded patients with a diagnosis of polycystic ovarian syndrome (who may sometimes be prescribed an oral anti-diabetic agent) unless they also received a diabetes diagnosis. Any patient with a diagnosis of gestational diabetes during the observation period was also excluded. Since less than 1% of all study patients initiated therapy on meglatinide, thiazolidinedione, or alpha-glucosidase inhibitors, we excluded them in the main analyses and focused on the vast majority of patients whose initial therapy was sulfonylurea or metformin. The resulting study sample consisted of 1906 patients.

### Measures

#### Patterns of medication use

We used four outcome measures to capture patients' patterns of medication use. First, we examined the time from diabetes diagnosis until the first prescription for oral antidiabetic medication. Second, among those with prescriptions, we examined primary adherence by measuring the time from first prescription for an oral antidiabetic drug until the patient filled the prescription. Third, we measured the time between first prescription and discontinuation of medication therapy (= 60 days without a refill following last day without medication). Lastly, we examined long-term adherence, defined as the monthly average rate of adherence, comparing dose available in each month through prior dispensings in relation to dose prescribed in each month over a 24-month follow-up from time of first prescription. To construct these adherence measures, we assessed refill-based medication adherence linking prescribing information from clinical encounter data and pharmacy claims for dispensed data. Standard refill-based medication adherence measures assume that days supply is equivalent to daily dose and, therefore, cannot distinguish between physician initiated changes in therapy and patient noncompliance. [24,25] Our measure is based on actual daily dose information from prescribing notes to determine intended daily dose. These then were linked to dispensed oral antidiabetic medication information which was allocated in daily amounts according to the most recent prescription until the supply was exhausted. A patient was considered to have discontinued therapy once 60 days had elapsed without any oral antidiabetic medication available. Patients had a strong financial incentive (e.g., smaller copayments) to fill prescriptions within the health care setting under study. Median co-payment levels for the most commonly used diabetes drugs were similar for blacks and whites (e.g., glyburide: median copay = $10). For each oral antidiabetic medication, a time-varying adherence measure was calculated as the milligrams dispensed divided by the amount prescribed per month to obtain a percentage of the prescribed among that was available for use. For patients taking more than one oral medication during follow-up, we calculated the combined average adherence per month.

#### Covariates

Our covariates were comprised of both baseline (defined as 12 months prior to the beginning of a patient's medication treatment) and time-varying covariates. Baseline covariates included gender, age, census block group-derived median neighborhood household income and average educational attainment level, body mass index (BMI), and average HbA1c level over the prior year. For time-varying covariates, we used a previously validated method [26] to calculate the presence and number of comorbidities per month by counting the number of non-diabetes medicines taken by each patient using the first eight digits of the American Hospital Formulary Services (AHFS) code. We also included as a control for severity of illness a monthly indicator of whether the patient had a diabetes-related hospitalization or emergency room visit (ICD-9 = 250.XX). To adjust for differences in patient involvement with the care system, we controlled for number of outpatient physician visits per month.

### Statistical analysis

We performed all statistical analyses using SAS V8.02 (Cary, NC SAS, Inc 2000). [27] Chi-square and t-test statistics were used to test race differences in demographic, clinical and utilization characteristics in the 12 months prior to first prescription of diabetes medication. Bivariate correlates of race (p < 0.2) were included as covariates in multivariate analyses.

Given the clinical differences in side-effects between sulfonylurea and metformin and the greater likelihood in discontinuation of metformin therapy due to its side-effects, we stratified all analyses by these two most commonly prescribed oral therapy groups. Patients on a combination of metformin and other oral antidiabetic medication were represented in the metformin group.

We conducted survival analyses to examine racial differences in the time to initiation and discontinuation of oral antidiabetic drug therapy. Within the oral antidiabetic drug therapy initiators, we produced unadjusted cumulative probability curves of time to first prescription since diagnosis, time to filling this first prescription, and time to discontinuation of medication use, all stratified by race. We fit Cox survival regression models, [27,28] with both fixed and time-dependent covariates, to estimate the hazard rate of blacks relative to whites for both initiation and discontinuation of oral antidiabetic medication. Patients were censored at the end of continuous enrollment or end of the 24-month follow-up.

For our final multivariate analyses, we estimated the effect of race on long-term adherence among patients for 24 months, allowing patients who discontinued use for more than 60 days but reinitiated therapy thereafter to contribute to these analyses. We excluded patients with fewer than 24 months of follow-up from the time of first prescription or who switched or augmented medication therapy with insulin during the 24-month follow-up. The resulting analytical sample for the adherence analyses included 1404 patients. Within therapy groups, we used log-linear models (generalized estimating equations) to estimate and test the difference in mean adherence rates for the two race groups at eight quarterly time intervals. In these models, we controlled for key demographic and clinical covariates as well as clinical sites serving disproportionately high numbers of black patients. Quarterly intervals were included in the model as dummy terms (with the initiation month of drug treatment as a referent). To test whether race differences varied across quarters, we used likelihood ratio tests. We also stratified these analyses by pre-treatment HbA1c group (average HbA1c levels >9.0%; between 7.0% and 9.0%; <7.0%) [29] to adjust for race differences in severity of diabetes prior to initiation of oral antidiabetic medication. This study was approved by the HPHC Institutional Review Board.

## Results

### Description of study cohort

As shown in Table 1, 74% of the 1906 patients in the analytic sample were white and 26% were black. Black patients were more likely to be female, younger, and to have poorer glycemic control at baseline, but had fewer non-diabetes medications prior to receiving their first prescription of oral antidiabetic drug therapy. Most patients started glucose self-monitoring within the 12 months prior to initiating an oral antidiabetic drug therapy, with more blacks initiating than whites. For both race groups, the majority of patients were prescribed a sulfonylurea as their first form of oral antidiabetic medication treatment.

**Table 1:** Racial differences in baseline* demographic and clinical status of diabetes patients (N = 1906)

	**Black** N = 498 (26.1%)	**White** N = 1408 (73.9%)
**Demographic Characteristics**		

Male	49.4	57.2^†^
Mean Age (SD)	45(10)	53(13)^†^

**Census-Derived SES Measures**		

Living in neighborhood with mean household income below poverty level	30.9	9.2^†^
Living in neighborhood where >75% residents do not understand spoken English	32.4	13.6^†^
Living in neighborhood where >75% residents do not have high school degree	5.6	0.8^†^

**Health Service Utilization**		

Mean # of MD Visits (SD)	3(2)	3(2)
Mean # of Lab Tests (SD)	2(1)	2(1)

**Clinical Characteristics**		

Glycemic Control^§^		
Good (<7.0%)	35.7	41.1
Moderate (7.0%–9.0%)	44.4	45.4
Poor (>9.0%)	19.9	13.6
Mean HbA1c Values^§ ^(SD)	7.8(1.6)	7.6(1.5)^†^
Body Mass Index (BMI)^||^		
Overweight (30–<40)	42.6	48.6
Obese (40+)	16.0	13.6
Mean BMI^|| ^(SD)	33.2(7.6)	32.8(6.6)

**Comorbidities**		

Any Diabetes-Related Hospitalizations	8.6	10.8
Any Diabetes-Related ER Visits	9.4	9.2
Monthly Mean # AHFS^¶ ^(SD)	1.9(0.9)	2.1(1.0)^†^

**Self-Management Practice**		

Initiation of Self-Monitoring Blood Glucose	70.7	59.6^†^
Initiation of Medication Therapy		
Sulfonylurea	86.7	85.8
Metformin (alone or in combination)	13.2	14.2

Data are expressed as % patients or mean (SD).

*Baseline = 12 months prior to first drug prescription, not including the month of first script.

^†^p <.05.

^§ ^Among those with a baseline HbA1c test (% with HbA1c data = Black: 34.3%; White: 41.3%)

^|| ^Among those with a baseline BMI (% with BMI data = Black: 49.0%; White: 52.1%).

^¶^AHFS = American Hospital Formulary Services.

### Effects of race on oral antidiabetic medication use over time

**Figure 1:**
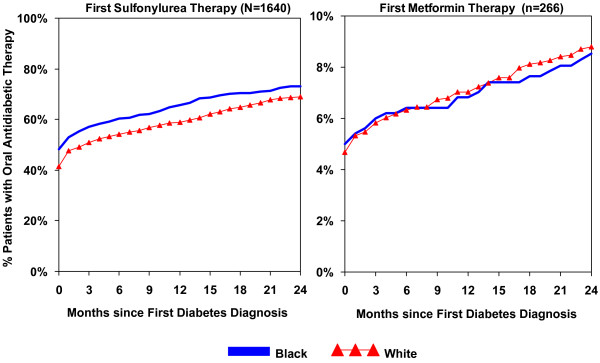
**Racial differences in cumulative initiation rates of prescribed oral antidiabetic therapy since first diabetes diagnosis**. Month 0 = Month of first diabetes diagnosis

Figure 1 shows race differences in cumulative rates of initiating an oral antidiabetic drug during 24 months following first diabetes diagnosis, stratified by sulfonylurea and metformin therapy groups. Among sulfonylurea therapy patients, the unadjusted rate of initiation of medication among blacks remained higher than among whites over time. However, after adjusting for demographic and clinical covariates, race no longer was a significant predictor of initiation among sulfonylurea therapy patients (Hazard Ratio, black versus white = 0.97, 95% Confidence Intervals: 0.6, 1.5). There were no race differences in initiation of medication among patients who were prescribed metformin as their initial therapy.

We found no black-white differences in primary adherence to medication for either drug therapy group. Over 90% of black and white patients filled their first prescription within 30 days. By the end of the 24-month follow-up, nearly all patients had filled their prescription at least once.

**Figure 2:**
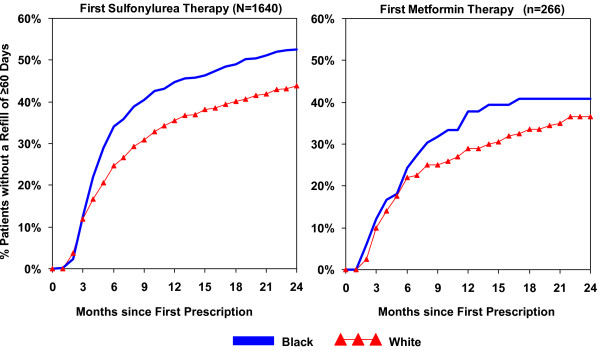
**Racial differences in cumulative rates of medication discontinuation among newly prescribed oral antidiabetic therapy patients**. Month 0 = Month of first medication prescription. Discontinuation = ≥ 60 days without available medication based on prescribed days' supply dispensed.

As shown in Figure 2, the cumulative rates of discontinuation of therapy among newly prescribed oral antidiabetic agent patients were consistently higher among blacks than whites within both the sulfonylurea and metformin groups. Substantial increases in medication discontinuation and notable differences between blacks and whites occurred by the end of the first six months of therapy (34% blacks vs. 25% whites). Discontinuation of medication use steadily increased thereafter for both drug and race groups, with fewer blacks continuing medication use than whites. By the end of follow-up, more than half of black patients had stopped medication use for more than 60 days, while 44% of whites had done so (p < 0.001). Unadjusted rates of discontinuation were similar among patients who received metformin. After controlling for key covariates (Table 2), blacks taking sulfonylurea medications had a rate of discontinuation 1.8 times higher than whites (95% CI: 1.2, 2.7). Blacks who initiated on a metformin medication had a rate of discontinuation 1.5 times that of whites, but after adjustment, this difference was not significant (95% CI: 0.4, 5.0).

**Table 2:** Adjusted predictors of discontinuation of first antidiabetic drug therapy during a 24-month follow-up period

	**Sulfonylurea (N = 1640)**		**Metformin (N = 266)**	
**Variable**	**Hazard Ratio**	**95% Confidence Interval**	**Hazard Ratio**	**95% Confidence Interval**

Black vs. White	1.8	1.2, 2.7^††^	1.5	0.4, 5.0
Male vs. Female	1.14	0.8, 1.6	1.6	0.6, 4.9
Age	1.02	1.01, 1.04^†^	1.0	0.9, 1.0
Low Income	1.0	0.7, 1.5	3.3	0.9, 11.5
< High School Education	0.5	0.1, 1.9	1.0	0.07, 14.1
HbA1c 7.0–9.0 vs. <7.0^§^	0.8	0.6, 1.3	0.4	0.05. 3.1
HbA1c > 9.0 vs. <7.0^§^	1.01	0.5, 1.9	4.6	0.5, 42.2
BMI (time-variant)	1.001	1.0, 1.03	1.0	0.9, 1.1
# AHFS^|| ^(time-variant)	0.9	0.8, 1.1	0.9	0.5, 1.7
# of MD Visits (time-variant)	1.0	0.8, 1.2	1.2	0.5, 2.5

Analysis adjusted for health centers with disproportionately high numbers of black patients

^†^p < .05; ^††^p < .01.

^§^Glycemic control based on baseline HbA1c levels.

^|| ^AHFS = American Hospital Formulary Services Dispensed.

### Effects of race on long-term adherence to oral antidiabetic drug therapy

**Figure 3:**
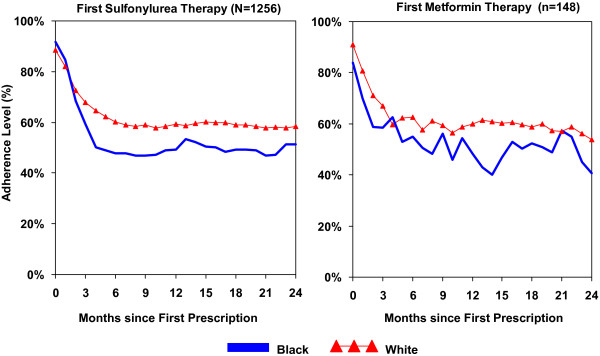
**Racial differences in long-term adherence among patients newly prescribed oral antidiabetic therapy**. Month 0 = Month of first medication prescription. Adherence (%) = (Total mgs available in month/Total mgs prescribed in month) * 100%.

Figure 3 compares black and white differences in long-term adherence to oral antidiabetic drug therapy during 24 months of follow-up. Levels of adherence were similar between sulfonylurea-treated and metformin-treated patients, with black-white differences in adherence apparent in both drug therapy groups. Among sulfonylurea therapy patients, both black and white patients showed drops in adherence levels within three months of initial medication therapy, with a more substantial decrease occurring among black patients. Although likely explained by high discontinuation rates of therapy by the third month as shown in Figure 2, this racial gap remained fairly constant thereafter; over time, blacks were taking on average 50% of their prescribed monthly dose of oral antidiabetic medication compared to an average of 60% of prescribed monthly dose among whites. Among the metformin therapy group, blacks were also consistently less adherent to medication than whites, with substantial drops occurring within three months of initiating medication for blacks and five months for whites.

After controlling for key covariates, blacks were consistently less adherent to sulfonylurea medication than whites, with significant black-white differences occurring within the second quarter of follow-up (Incidence Rate Ratio, blacks versus whites: 0.86; 95% CI: 0.78, 0.96, Table 3). Race differences decreased in the third quarter and remained relatively constant (IRR = 0.90–0.96) in subsequent quarters. Among patients on metformin therapy, there was a significant race difference in non-adherence between blacks and whites across quarters (IRR, blacks vs. whites: 0.79; 95% CI: 0.63, 0.99), but the magnitude of this difference fluctuated somewhat over the follow-up period. All rates in Table 3 reflect the main effect for race and quarter effects.

**Table 3:** Racial differences in average adherence to oral antidiabetic medication over a 24-month follow-up period

	**Sulfonylurea (n = 1256)**		**Metformin (n = 148)**	
**Time Since First Prescription**	**Incidence Rate Ratio**	**95% Confidence Interval**	**Incidence Rate Ratio**	**95% Confidence Interval**

Across All Quarters (Blacks vs. Whites)	0.96	0.89, 1.05	0.79	0.63, 0.99^†^

Within Quarters (Blacks vs. Whites)				
Quarter 1	1.00	0.92,1.09	0.78	0.80, 1.00
Quarter 2	0.86	0.78, 0.96^†^	0.86	0.65, 1.13
Quarter 3	0.90	0.80, 1.00	0.92	0.69, 1.21
Quarter 4	0.91	0.81, 1.02	0.72	0.49, 1.06
Quarter 5	0.94	0.84, 1.05	0.63	0.41, 0.96^†^
Quarter 6	0.92	0.81, 1.03	0.83	0.59, 1.17
Quarter 7	0.92	0.81, 1.05	0.69	0.45, 1.06
Quarter 8	0.96	0.84, 1.04	0.59	0.35, 0.97^†^

Adjusted for age, gender, neighborhood socioeconomic status, body mass index, any HbA1c test, baseline glycemic level, number of MD visits, and number of comorbidities, health centers with disproportionately high numbers of black patients; Referent Group: initiation month of treatment group; All rates reflect both main effect of race and quarter effects.

^†^p < .05

## Discussion

In this longitudinal investigation of adherence to oral antidiabetic medication in a large, fully insured HMO diabetes population, we found no racial differences in initiation of treatment or in primary adherence (time until first prescription filled) among newly diagnosed diabetes patients. However, rates of discontinuation in medication use varied by race, with blacks discontinuing sooner and at higher rates than whites, particularly among patients initiating sulfonylurea therapy. Re-initiation of therapy after discontinuing for more than 60 days did not appear to show a substantial increase in adherence levels for either race group over time. In concordance with our hypothesis, blacks were consistently less adherent to oral antidiabetic drug therapy than whites over time.

Our findings agree with previous studies reporting that blacks have lower adherence to oral antidiabetic medication.[14] However, our longitudinal study is unique in that we linked prescribing information to dispensing data in order to build a more precise measure of daily and monthly average adherence, which is important when studying a population whose health status and medical treatment change frequently. Furthermore, the use of multiple years of follow-up among a relatively large number of fully insured, HMO population of black and white diabetes patients enabled us to identify when adherence levels began to drop during the course of therapy, and when levels of adherence for blacks and whites began to diverge. The ability to identify time points when divergence with respect to medication adherence occurs is important information for clinicians working to support patients' adherence over time and for developing more effective system-level interventions.

While our findings demonstrate persistent racial differences in adherence levels for patients on sulfonylurea or metformin therapies, racial differences in patterns of medication use varied by drug therapy group. For those taking sulfonylureas, the largest reductions in adherence and the separation in adherence levels between black and whites occurred early in treatment (quarter 2–3 following initiation of therapy); racial differences appeared to decline somewhat thereafter. This modest narrowing of race differences over time may result from patients reinitiating their medication therapy after early discontinuation. Among metformin patients, race differences in adherence, while somewhat inconsistent, appear to increase over time. Additional research is needed to understand longitudinal variations in adherence, timing of initiation of insulin or re-initiation of oral antidiabetic drug use following discontinuation, and racial differences in these behaviors.

In a recent study, [17] we found that even within a managed care setting where equal access to services and high quality of care are provided, some blacks still had poorer glycemic control than whites. Our current study demonstrates that adherence to oral antidiabetic drug therapy is lower among blacks than whites, and suggest that these persistent racial differences may account for part of the observed racial differences in glycemic control found in that previous study. However, other unmeasured patient-level factors may help to explain these disparities in medication adherence and diabetes outcomes. For example, racial and ethnic minorities with diabetes may face greater barriers to medication adherence due to lower socioeconomic status, poorer health literacy, higher rates of distrust of providers and the health care system, concerns about the efficacy of medications or their side effects, cost-related underuse, and other psychosocial factors. [12,13] We also did not examine other potential determinants such as differences in regimen complexity, provider-patient communication, clinical inertia, variations in prescribing patterns, or provider perceptions about the benefits of tight adherence.

This study has additional potential limitations worthy of discussion. First, because race was determined from clinician reports, misclassification may have occurred. However, assessments of white and black race from these sources have been shown to be highly reliable. [22,23] Race/ethnicity may also serve as a proxy for a myriad of cultural and psychosocial factors that can influence patient medication use. [30] A key advantage to our study setting is that several potential race-related differences in economic factors (e.g., comparable co-payments) and system-based barriers to care (e.g., higher quality of care and access to services) have been minimized. To better isolate the effect of race and capture differences in some of the cultural and psychosocial factors associated with medication use, we controlled for neighborhood educational attainment and income, but we did not have measures of other cultural and psychosocial factors that may be closely correlated with race. Therefore, any racial differences observed in our analyses may in part represent unmeasured socio-cultural as well as biological constructs. [31]

We controlled for a number of other potentially race-related barriers to adherence, such as the number of physician visits per month. However, by doing so, we may have over-controlled for racial disparities, such that we underestimate the effect of race on adherence. For example, if differences in adherence are due to blacks' use of fewer services or receipt of less patient education, controlling for those factors may reduce estimates of the impact of race on adherence behavior. For future work, a focus on the volume and nature of patient interactions with the health care system may enable us to identify additional determinants of racial disparities and avenues for intervention.

The rates of missing data for HbA1c and BMI in Table 1 are high for this study setting. However, these high rates are driven by our definition of the baseline period, which does not include the month of the first prescription. Therefore, the low rate of capture is an artifact of the study design and not an indication of the quality of the data or of the care this study setting provides.

The age of our data represents a decade when sulfonylureas were prescribed over metformin as the first line of treatment contrary to ADA recommendation. However, over time, metformin has become more commonly prescribed as the initial oral therapy over sulfonylureas, particularly among obese patients. While the rate of initiating sulfonylureas and metformin reported in this study may not represent the current prescribing behavior of providers, self-management guidelines have not changed since our study data and the implications of these study findings remain very relevant to current medical practice.

Finally, our findings are restricted to a single multi-specialty care group in Massachusetts. While we cannot generalize our findings to non-managed care settings, the large proportion of black patients enrolled at HVMA allows for comparison to other studies of racial/ethnic differences in diabetes self-management in similar settings.

## Conclusion

Racial differences in adherence to oral antidiabetic drug therapy persist over time within a health system that provides uniform access to services and high quality of care for diabetes patients. While differences do not appear in initiation of medication, racial gaps are quickly evident in discontinuation and adherence over time. Early and continued emphasis on sustaining medication use after initiation of medication may lead to improved adherence to antidiabetic medication. Aggressive, culturally-specific promotion of adherence among blacks or implementation of a patient-centered approach to identify barriers to adherence and provide self-care supports during early treatment may potentially reduce persistent racial differences in self-management practice and clinical outcomes.

## Competing interests

The authors declare no competing interests including specific financial interests and relationships and affiliations relevant to the subject of this manuscript.

## Authors' contributions

Substantial contributions to conception and design, or acquisition of data, or analysis and interpretation of data (CMT, ASA, FZ, SBS, JBM, JDP, DRD). Substantial involvement in drafting the manuscript or revising it critically for important intellectual content (CMT, ASA, SBS, JBM, JDP, DRD). Provision of final approval of publishable version (CMT, ASA, FZ, SBS, JBM, JDP, DRD)

## Pre-publication history

The pre-publication history for this paper can be accessed here:



## References

[B1] (2008). Economic costs of diabetes in the U.S. in 2007. Diabetes Care.

[B2] Harris MI, Eastman RC, Cowie CC, Flegal KM, Eberhardt MS (1999). Racial and ethnic differences in glycemic control of adults with type 2 diabetes. Diabetes Care.

[B3] Carter JS, Pugh JA, Monterrosa A (1996). Non-insulin-dependent diabetes mellitus in minorities in the United States. Ann Intern Med.

[B4] Gerstein HC, Miller ME, Byington RP, Goff DC, Bigger JT, Buse JB, Cushman WC, Genuth S, Ismail-Beigi F, Grimm RH, Probstfield JL, Simons-Morton DG, Friedewald WT (2008). Effects of intensive glucose lowering in type 2 diabetes. N Engl J Med.

[B5] Holman RR, Paul SK, Bethel MA, Matthews DR, Neil HA (2008). 10-year follow-up of intensive glucose control in type 2 diabetes. N Engl J Med.

[B6] Patel A, MacMahon S, Chalmers J, Neal B, Billot L, Woodward M, Marre M, Cooper M, Glasziou P, Grobbee D, Hamet P, Harrap S, Heller S, Liu L, Mancia G, Mogensen CE, Pan C, Poulter N, Rodgers A, Williams B, Bompoint S, de Galan BE, Joshi R, Travert F (2008). Intensive blood glucose control and vascular outcomes in patients with type 2 diabetes. N Engl J Med.

[B7] Gaede P, Lund-Andersen H, Parving HH, Pedersen O (2008). Effect of a multifactorial intervention on mortality in type 2 diabetes. N Engl J Med.

[B8] Boccuzzi SJ, Wogen J, Fox J, Sung JC, Shah AB, Kim J (2001). Utilization of oral hypoglycemic agents in a drug-insured U.S. population. Diabetes Care.

[B9] Donnan PT, MacDonald TM, Morris AD (2002). Adherence to prescribed oral hypoglycaemic medication in a population of patients with Type 2 diabetes: a retrospective cohort study. Diabet Med.

[B10] Morningstar BA, Sketris IS, Kephart GC, Sclar DA (2002). Variation in pharmacy prescription refill adherence measures by type of oral antihyperglycaemic drug therapy in seniors in Nova Scotia, Canada. J Clin Pharm Ther.

[B11] Melikian C, White TJ, Vanderplas A, Dezii CM, Chang E (2002). Adherence to oral antidiabetic therapy in a managed care organization: a comparison of monotherapy, combination therapy, and fixed-dose combination therapy. Clin Ther.

[B12] Auslander WF, Thompson S, Dreitzer D, White NH, Santiago JV (1997). Disparity in glycemic control and adherence between African-American and Caucasian youths with diabetes. Family and community contexts. Diabetes Care.

[B13] Cowie CC, Harris MI (1997). Ambulatory medical care for non-Hispanic whites, African-Americans, and Mexican-Americans with NIDDM in the U.S. Diabetes Care.

[B14] Heisler M, Faul JD, Hayward RA, Langa KM, Blaum C, Weir D (2007). Mechanisms for racial and ethnic disparities in glycemic control in middle-aged and older Americans in the health and retirement study. Arch Intern Med.

[B15] Glasgow RE, Hampson SE, Strycker LA, Ruggiero L (1997). Personal-model beliefs and social-environmental barriers related to diabetes self-management. Diabetes Care.

[B16] Karter AJ, Ferrara A, Darbinian JA, Ackerson LM, Selby JV (2000). Self-monitoring of blood glucose: language and financial barriers in a managed care population with diabetes. Diabetes Care.

[B17] Adams AS, Zhang F, Mah C, Grant RW, Kleinman K, Meigs JB, Ross-Degnan D (2005). Race differences in long-term diabetes management in an HMO. Diabetes Care.

[B18] Trinacty CM, Adams AS, Soumerai SB, Zhang F, Meigs JB, Piette JD, Ross-Degnan D (2007). Racial differences in long-term self-monitoring practice among newly drug-treated diabetes patients in an HMO. J Gen Intern Med.

[B19] Mah CA, Soumerai SB, Adams AS, Ross-Degnan D (2006). Racial differences in impact of coverage on diabetes self-monitoring in a health maintenance organization. Med Care.

[B20] Jaber LA, Halapy H, Fernet M, Tummalapalli S, Diwakaran H (1996). Evaluation of a pharmaceutical care model on diabetes management. Ann Pharmacother.

[B21] Ruggiero L, Glasgow R, Dryfoos JM, Rossi JS, Prochaska JO, Orleans CT, Prokhorov AV, Rossi SR, Greene GW, Reed GR, Kelly K, Chobanian L, Johnson S (1997). Diabetes self-management. Self-reported recommendations and patterns in a large population. Diabetes Care.

[B22] Choo PW, Rand CS, Inui TS, Lee ML, Cain E, Cordeiro-Breault M, Canning C, Platt R (1999). Validation of patient reports, automated pharmacy records, and pill counts with electronic monitoring of adherence to antihypertensive therapy. Med Care.

[B23] Chan KA, Davis RL, Gunter MJ, Gurwitz JH, Herrinton LJ, Nelson WW, Raebel MA, Roblin DW, Smith DH, Platt R (2005). HMO Research Network. Pharmacoepidemiology.

[B24] Steiner JF, Prochazka AV (1997). The assessment of refill compliance using pharmacy records: methods, validity, and applications. J Clin Epidemiol.

[B25] Andrade SE, Kahler KH, Frech F, Chan KA (2006). Methods for evaluation of medication adherence and persistence using automated databases. Pharmacoepidemiol Drug Saf.

[B26] Schneeweiss S, Seeger JD, Maclure M, Wang PS, Avorn J, Glynn RJ (2001). Performance of comorbidity scores to control for confounding in epidemiologic studies using claims data. Am J Epidemiol.

[B27] SAS Institute Inc (1999). SAS/STAT® User's Guide, Version 8.

[B28] Allison PD (1995). Survival Analysis Using the SAS System: A Practical Guide.

[B29] Rosival V (2003). Clarification of statements in 2003 clinical practice recommendations. Diabetes Care.

[B30] Meier JL, Swislocki AL, Lopez JR, Noth RH, Bartlebaugh P, Siegel D (2002). Reduction in self-monitoring of blood glucose in persons with type 2 diabetes results in cost savings and no change in glycemic control. Am J Manag Care.

[B31] Williams DR (1994). The concept of race in Health Services Research: 1966 to 1990. Health Serv Res.

